# Prevalence and risk factors of three curable sexually transmitted infections among women in Nairobi, Kenya

**DOI:** 10.1186/s13104-016-1990-x

**Published:** 2016-03-29

**Authors:** Anne Njeri Maina, Joshua Kimani, Omu Anzala

**Affiliations:** Department of Medical Microbiology, University of Nairobi, P.O BOX 19676-00202, Nairobi, Kenya; Kenya AIDS Control Project, University of Manitoba/Nairobi Collaborative Research Group, P.O BOX 19676-00202, Nairobi, Kenya; KAVI Institute of Clinical Research, University of Nairobi, P.O BOX 19676-00202, Nairobi, Kenya

**Keywords:** Prevalence, Sexually transmitted infections, Women, Family planning clinic, Kenya

## Abstract

**Background:**

Sexually transmitted infections (STIs) are a major public health problem, especially in developing countries. The complications of untreated STIs in the female genital tract and their role in adverse pregnancy and perinatal outcomes have been well documented. The prevalence of STIs in Kenya among women in the general population has not been extensively studied and there is a lack of guidelines for screening of non-pregnant women. Knowledge of the prevalence of curable STIs among this population can provide a basis for integrating STI screening in family planning clinics.

**Methods:**

A cross-sectional study was conducted between May and September 2013 at the family planning (FP) clinic at Kenyatta National Hospital (KNH) in Nairobi, Kenya. A total of 261 participants aged 18–49 years were enrolled; with data from 249 participants being analysed. An interviewer-administered questionnaire was used to gather socio-demographic data and assess for risk factors. Each participant was screened for *Trichomonas vaginalis* (TV*)* by wet mount microscopy; *Neisseria gonorrhoeae* (GC) by culture and *Chlamydia trachomatis* (CT) by PCR.

**Results:**

The prevalence of CT was 13 % (33/249), TV 0.4 % (1/249) and GC 0 % (0/249). All the infected women reported having had only one sexual partner in the previous 1 year. The age group prevalence for CT was highest in the 25–29 years age group (21 %). The syndromic approach to the management of STIs showed a low specificity (vaginal discharge, 65.7 %; lower abdominal pain, 60.6 %) and positive predictive value (vaginal discharge, 14 %; lower abdominal pain, 11.5 %) for the two commonly used symptoms when compared to the gold standard of CT PCR.

**Conclusion:**

A high prevalence of CT was identified among women attending the FP Clinic at KNH. The study reinforces the need to implement regular screening for STIs among FP clinic attendants. It also reveals the need to review the usage of the syndromic approach for the management of STIs.

## Background

According to the 2008 WHO prevalence estimates for sexually transmitted infections (STIs), women in the African region had a higher prevalence than men for *Neisseria gonorrhoeae* (GC), *Chlamydia trachomatis* (CT) and *Trichomonas vaginalis* (TV) at 2.3, 2.6 and 20.2 % respectively [1]. Despite a majority of cases of these curable STIs being asymptomatic [2], untreated STIs have been linked with complications such as adverse pregnancy and perinatal outcomes [3]; chronic pelvic illnesses [4] and increased risk of HIV acquisition [5]. Screening for STIs among women is therefore an important way of identifying asymptomatic infections before any complications can arise. Indeed, studies have shown that screening for STIs reduces their sequelae [6].

Most of the recent information about the prevalence of STIs among women in Kenya has been from studies done among high-risk individuals [7] and special categories like pregnant women [8]. There is however a gap in information about the prevalence of STIs among women in the general population. This is largely because the surveillance of STIs, although recommended [9], has not been well implemented; with the major focus in Kenya in the last 10–15 years being on HIV [10]. This is further complicated by the fact that no guidelines exist for the screening of non-pregnant women for curable STIs.

In Kenya, the syndromic approach to treatment of genital infections is still used in public health facilities. This approach has however been shown to have little effect on the prevalence of STIs that are mostly asymptomatic [11]. Further, studies have demonstrated the high proportion of infected individuals with no reported symptoms who would provide a reservoir for infection and reinfection of susceptible individuals [2].

This study was therefore undertaken to determine the prevalence and the risk factors for *C. trachomatis*, *N. gonorrhoeae* and *T. vaginalis* in women aged 18–49 years attending the Family Planning Clinic at Kenyatta National Hospital in Nairobi, Kenya.

## Methods

We conducted a prospective cross-sectional study at Kenyatta National Hospital’s (KNH) family planning (FP) clinic in Nairobi, Kenya between May and September 2013. The KNH FP clinic, which offers contraceptive services, runs daily from Monday to Friday between 9 a.m. and 4 p.m. and has an average attendance of five clients per day. All women aged 18–49 years attending the FP clinic during the study period were invited to participate in the study. Recruitment was done throughout the day by either the principal investigator or study nurses who also obtained written informed consent. Recruitment continued until the desired sample size was achieved. Inclusion criteria were age 18–49 years and willingness to participate in the study. Exclusion criteria were women who declined to give informed consent, those who had been treated for STIs and/or had been on broad spectrum antimicrobial agents in the preceding 3 weeks.

After informed consent had been obtained, a study nurse administered a questionnaire covering demographic data, sexual and symptomatic history. Two endocervical samples were then collected from each participant and transported to the Department of Medical Microbiology, University of Nairobi within 5 minutes. The following tests were carried out: saline wet mounts were examined at 40× power for the presence of motile trichomonads, culture for *N. gonorrhoeae* was carried out on modified Thayer–Martin media and PCR for *C. trachomatis* (GenoQuick CT, Hain Lifescience, Nehren Germany) according to the manufacturer’s instructions.

Data was entered into Microsoft Excel and analysed using SPSS version 17.0. Chi square and Fisher’s exact tests were used to determine univariate associations with prevalent *C. trachomatis* infection. A p value of <0.05 was considered to be statistically significant. This study was approved by the Kenyatta National Hospital/University of Nairobi Ethical Review Committee (Ref: KNH-ERC/A/96).

## Results

A total of 261 participants, attending routine family planning clinic, were recruited consecutively during the May to September 2013 study period. Data from 12 participants was excluded from analysis: two had not been sexually active for more than 4 years (the two were prisoners preparing for release to their families), while 10 were discovered to have used broad-spectrum antimicrobial agents within the previous 2 weeks prior to the study. Data from 249 participants was therefore analysed.

The median age of the participants was 37 years (range: 20–49 years). Majority of the participants were married (85 %) and 73 % had a level of education of secondary school and above. The demographic characteristics of the participants are summarised in Table 1.

**Table 1:** Characteristics of the study participants

	*n*	%
Age group		
<25	13	5
25–29	28	11
30–34	56	23
35–39	67	27
40–44	48	19
>45–49	37	15
Marital status		
Currently married	211	85
Not currently married	38	15
Education		
Primary and below	67	27
Above primary	182	73
Occupation		
Paid employment	83	33
Self employed	127	51
Unemployed	39	16
Coitarche		
≤20 years	160	64
>20 years	78	31
Missing	11	5
Treated STIs in last 3 months		
No	224	90
Yes	25	10
New sexual partner in the last 3 months		
No	240	96
Yes	9	4
Number of sexual partners in last 1 year		
0	11	4
1	226	91
≥2	12	5

The median age of sexual debut was 20 years (range: 5–33 years). A majority of the participants (91 %) had only 1 sexual partner in the preceding 1 year while 4 and 0.4 % had 2 and 3 sexual partners respectively in the preceding 1 year. Fifteen percentage of participants reported condom use during their last sex act. Treatment for STIs in the previous 3 months was reported by 10 % of participants.

The prevalence of CT was 13 % (33/249) (95 % CI 9.5, 17.9), and TV 0.4 % (1/249). A co-infection of CT and TV was found in 0.4 % (1/249) participant. No participants (0 %; 0/249) were found to have GC.

The frequency of CT was highest among women aged 25–29 years and lowest among those 45–49 years as shown in Table 2.

**Table 2:** Characteristics of CT positive participants

Characteristics	Chlamydia infections/n	Prevalence (%)	P value
Age group			
<25	1/13	8	
25–29	6/28	21	
30–34	6/56	11	
35–39	10/67	15	
40–44	8/48	17	
45–49	2/37	5	
Marital status			
Married	29/211	14	0.59
Not married	4/38	11	
Education level			
Primary and below	11/67	16	0.37
Above primary	22/182	12	
Occupation			
Paid employment	11/83	13	0.9
Self employed	16/127	13	
Unemployed	6/39	15	
Coitarche			
≤20 years	21/160	13	0.95
>20 years	10/78	13	
Treated STIs			
No	32/224	14	0.22
Yes	1/25	4	

A majority, 30/33 (88 %), of CT positive participants were married with all of them having had only one sexual partner in the previous one year although on further analysis of those currently married and those not currently married, no statistical significance was found (p = 0.59). The characteristics of CT positive participants are summarised in Table 2.

The most commonly reported symptoms among the CT infected participants were vaginal discharge (36 %) and lower abdominal pain (33 %). However, 21/33 (64 %) of those who tested positive for CT did not report any vaginal discharge and 22/33 (67 %) had no lower abdominal pain.

Using the CT PCR used in the current study as the gold standard, the sensitivity, specificity and positive predictive values (PPV) for the different symptoms used in the syndromic management of STIs was calculated as shown in Table 3.

**Table 3:** Sensitivity, specificity and positive predictive value (PPV) of symptoms using CT PCR as the standard

	Sensitivity (%)	Specificity (%)	PPV (%)
Vaginal discharge	36	66	14
Lower abdominal pain	33	61	12
Vaginal itch	24	76	13
Pain on micturition	3	83	5
Intermenstrual bleeding	3	88	4
Bleeding after sexual intercourse	12	93	21

All the infected participants were informed of their results and requested to come to the FP clinic where relevant treatment was prescribed. They were also advised to disclose the results to their sexual partners. Thirty one out of the thirty three infected participants showed up at the clinic for treatment. Two participants showed up with their partners where free screening was offered to the partners by the Principal Investigator. One participant had received syndromic management before the CT PCR results were released to her. One participant declined medication.

## Discussion

The prevalence of *C. trachomatis* (13 %) found in this study is comparable with that which has been reported in previous published studies from Kenya involving women. A CT prevalence of 12 % was found among women using intrauterine contraceptive devices at the KNH FP clinic between 1984 and 1986 [12]; Kohli et al. [13] found a prevalence of 6 % among women attending outpatient clinics in Nairobi; 4 % was found among HIV-1 infected pregnant women and 4 % among women attending an STD referral clinic [14, 15] respectively. This indicates that the prevalence of *C. trachomatis* is still significant in the female population and introduction of routine screening procedures at FP clinics would help in reducing the burden of the disease among women.

However, the prevalence of 0 and 0.4 % for *N. gonorrhoeae* and *T. vaginalis* respectively is lower than what has been previously published in studies in which similar laboratory procedures were used. Marx et al. [14] found a TV prevalence of 16 % and a GC prevalence of 2 % among HIV-1 infected pregnant women in Nairobi; Fonck et al. [15] found a high prevalence of 23 % for TV and 7 % for GC among women with complaints of vaginal discharge attending an STD referral clinic in Nairobi while Daly et al. [16] found a GC prevalence of 3.2 % and a TV prevalence of 5.2 % among women attending FP clinic in Nairobi. It was not clear why the prevalence of TV in this study was low as most studies have shown that a higher prevalence of TV is found in women above the age of 30 years, which was the age for a majority of participants in this study. The low GC and TV prevalence could be explained by the fact that molecular methods were used for the detection of CT while GC was detected by culture while TV was detected by wet mount whose sensitivity varies from 38 to 82 % [17, 18].

All the women that were diagnosed with CT and TV reported having had only one sexual partner in the preceding 1 year and only 1/33 (3 %) reported a new sexual partner in the 3 months prior to the study. These findings therefore suggest that the women were likely to have been infected by their regular partner. It further stresses the importance of partner notification, testing and treatment as well as addressing the significant impact of the transmission between partners and the importance of directing prevention campaigns towards reducing infection from regular sexual partners.

The high proportion of asymptomatic participants who tested positive for *C. trachomatis* (64 % for vaginal discharge and 67 % for lower abdominal pain) corroborates findings in other studies and further stresses the importance of the aetiologic approach as opposed to the syndromic approach of managing STIs. However, due to lack of adequate laboratory infrastructure in the country, the WHO recommended syndromic approach for the management of STIs is utilised [19]. The syndromic management approach is based on the identification of consistent groups of symptoms and easily recognised signs and syndromes and the provision of treatment that will deal with the majority of or the most serious organisms responsible for producing a syndrome [20]. In the current study, evaluation of the diagnosis accuracy of the most commonly used symptoms in the syndromic approach using the CT PCR as the gold standard was done. The evaluation showed a sensitivity of 36 and 33 %, a specificity of 66 and 61 % and a positive predictive value of 14 and 12 % for vaginal discharge and lower abdominal pain respectively which are the most commonly used symptoms in the syndromic approach of managing STIs. This is comparable with what has been found in other studies: Detels R [2] found that over 80 % of the participants, who were drawn from five countries, were asymptomatic and that the positive predictive value of urethral or vaginal discharge was 58 %; Fonck et al. [14] found that the algorithm in use in Kenya as national policy had a sensitivity of 42 % and a specificity of 63 % for the detection of GC or CT and thus failed to discriminate between infected and uninfected women. This complicates identification and treatment of infected individuals and it also provides a large pool of asymptomatic transmitters since individuals who have no symptoms are unlikely to seek testing and treatment. The findings from this study therefore lead to the conclusion that the syndromic approach to the management of STIs leads to an underestimation of the proportion of infected individuals, as a large majority tend to be asymptomatic. The aetiologic approach to management for symptomatic patients is therefore recommended in addition to screening in order to detect asymptomatic individuals.

Only 13/249 (5.2 %) participants were aged between 18 and 24 years. This could be explained by the fact the KNH FP clinic charges for its FP services which could put them out of reach for women in this age group as a majority of them may be unemployed and/or in colleges and universities. The study therefore could have missed an age group with a higher burden of infection or more risk factors for the acquisition of STIs. The use of an interviewer-administered questionnaire to gather sensitive information on sexual history could have led to underreporting of sexual risk behaviours. The findings therefore cannot easily be generalized to women in Kenya who may have different sociodemographic characteristics and risk factors. A study involving women in the adolescent age group is therefore recommended.

## Conclusions

The prevalence of *C. trachomatis* is significantly high among women in the general population. There is need to implement routine screening tests for CT and other STIs in FP clinics to aid in identifying asymptomatic cases and treating them before any sequelae arise. This study provides further evidence of the need to implement aetiologic management of STIs and the significance of directing preventive messages to individuals with regular sexual partners. There is an urgent need for studies designed to investigate the prevalence and risk factors of STIs among women aged 18–24 years in Kenya.

## Abbreviations

CDC: Centres for Disease Control
CI: confidence interval
CT: *Chlamydia trachomatis*
FP: family planning
GC: *Neisseria gonorrhoeae*
HIV: Human Immunodeficiency Virus
KNH: Kenyatta National Hospital
PCR: polymerase chain reaction
PPV: positive predictive value
SPSS: statistical package for social scientists
STD: sexually transmitted diseases
STI: sexually transmitted infections
TV: *Trichomonas vaginalis*
WHO: World Health Organization
